# NO Time to Die: Nitric Oxide’s Ongoing Relevance in Mental Disorders

**DOI:** 10.1016/j.bpsgos.2025.100611

**Published:** 2025-10-23

**Authors:** Florian Freudenberg

**Affiliations:** Department of Psychiatry, Psychosomatic Medicine and Psychotherapy, University Hospital Frankfurt, Goethe University Frankfurt, Frankfurt, Germany

In 1992, the journal *Science* named nitric oxide (NO) “the Molecule of the Year.” In their announcement, the journal not only highlighted the possibilities for puns with this gaseous molecule’s chemical formula but also emphasized the versatile role of NO in biological mechanisms and in health and disease (1). Endogenous NO is synthesized by one of 3 nitric oxide synthase (NOS) isoforms: neuronal NOS (nNOS), inducible NOS (iNOS), and endothelial NOS. Despite their names implying restricted expression, one or more NOS isoforms can be found in many different cell types, enabling NO production in a large number of organs and tissues. Among its many physiological functions are vascular tone and homeostasis, immune regulation, and neurotransmission (2).

With the identification of nNOS as a neuronal producer of NO and the cloning of the *NOS1* gene (which encodes nNOS), the scientific interest for NO in brain function and disease rapidly increased. In the brain, NO serves as an important signaling molecule that affects neurotransmitter release, synaptic plasticity, and learning and memory. Consistent with these roles, NO signaling and homeostasis have been associated with several disorders of the brain, including mental disorders. Indeed, substantial evidence ranging from preclinical studies to human genetics and neuroimaging has linked NO and nNOS and/or its gene *NOS1* to schizophrenia, bipolar disorder, major depressive disorder (MDD), attention-deficit/hyperactivity disorder (ADHD), and other mental illnesses (3).

In new work published in *Biological Psychiatry Global Open Science*, Vareltzoglou *et al.* (4) summarize converging evidence for the involvement of DDAH (dimethylarginine dimethylaminohydrolase) proteins in mental disorder pathology and progression. Two DDAH isoforms with distinct mechanisms have been described. DDAH1 is an enzyme that catalyzes the hydrolysis of the endogenous NOS inhibitor ADMA (asymmetric dimethylarginine) to dimethylamine and citrulline. In contrast, DDAH2 does not hydrolyze ADMA but may affect NO signaling indirectly (5). In their narrative review, the authors make a convincing case for DDAH proteins in a range of mental disorders including MDD, bipolar disorder, posttraumatic stress disorder, schizophrenia, and ADHD. They compiled evidence from protein and gene expression studies in postmortem tissue of patients, genetic analyses of *DDAH1* and *DDAH2* variants, findings from preclinical studies, as well as measurements of serum/plasma ADMA and NO levels. Most studies reported alterations in these measures, although not always in the same direction. For example, the usually observed increase in ADMA levels in patients with mental disorders is contrasted by some studies finding unchanged or reduced levels of ADMA. In the review, the authors discuss these discrepancies as possibly being caused by differences in psychiatric and somatic comorbidities and by disease stage and severity. However, the authors conclude that DDAH-related measures may serve as promising biomarkers, and the DDAH/ADMA/NOS pathway may have therapeutic potential (4).

The findings presented on DDAH fit well into the vast literature on NO-related changes in mental disorder pathology. As noted, nNOS has been directly linked to several mental disorders and so have other proteins that are associated with nNOS. In neurons, nNOS is anchored to the postsynaptic density of glutamatergic synapses via interaction with PSD-93/PSD-95. This brings nNOS in proximity to NMDA receptors, a class of ionotropic glutamate receptors that can conduct calcium ions, which are required for nNOS-mediated NO synthesis. NO is a small, membrane-permeant gas that can freely diffuse across membranes. Thus, this molecule can act not only as a retrograde messenger that presynaptically leads to the facilitation of transmitter release through activation of soluble guanylate cyclase (sGC) but also in an sGC-independent manner. In addition, NO can act postsynaptically or on cells and synapses in proximity to its site of synthesis, although its reach is limited by its relative instability (2,3). Another mechanism by which nNOS can affect proteins in its vicinity is through S-nitrosylation (i.e., the addition a nitrosyl group to the thiol side group of cysteines). Targets for S-nitrosylation by nNOS include not only the NMDA receptor, leading to feedback inhibition, but also a number of other targets, such as RasD1, one of the known downstream mediators of nNOS activity.

Another critical component of the NO pathway called NOS1AP (nitric oxide synthase 1 adapter protein; previously named CAPON) directly interacts with nNOS and at the same time contains interaction sites for other nNOS mediators. Thus, NOS1AP serves as a scaffolding protein that, among others, enables NMDA receptor–nNOS-mediated activation of downstream pathways such as the p38MAPK and possibly also the JNK pathway. Moreover, the nitrosylation of RasD1 is mediated via interaction with NOS1AP. Notably, NOS1AP itself is a protein for which diverse evidence for an involvement in a range of mental disorders has been published, and for the past decade, this protein has been the primary research target of our research group. Our work and that of others on NO/nNOS/NOS1AP mechanisms, such as those described by Vareltzoglou *et al.* (4), have underscored both their pathological relevance and the complexity of their interactions.

As also emphasized by Vareltzoglou *et al.* (4), DDAH has been linked to cardiovascular disease. This is consistent with the well-established role of NO signaling in cardiovascular physiology (6), and in fact, several of the NO pathway proteins, including nNOS and NOS1AP, have been linked to cardiovascular disease. Thus, deficits in NO signaling may be one of the factors explaining the strong co-occurrence of negative cardiovascular outcomes with many mental disorders.

Despite extensive evidence implicating NO pathways, clinical translation has been difficult. One notable exception is the NOS/sGC-inhibitor methylene blue, which has shown efficacy in mood disorders. However, it is unclear whether its clinical efficacy can be (solely) attributed to the effect on the NO-pathway because methylene blue has also been shown to block GABA_A_ (gamma-aminobutyric acid A) receptors and monoamine oxidase A and to reduce inflammation (7). Other NO modulators have shown limited and inconsistent clinical benefits to date. For example, early promising findings for the NO donor sodium nitroprusside were later met by mixed results indicating unclear clinical benefit. Likely reasons for this include the comparatively small and heterogeneous samples in most trials and the high potential for drug concentrations outside the therapeutic window (8). Another important reason for the limited clinical success is the potential for side effects. For example, as alluded to above, NO signaling is an important contributor to cardiovascular mechanisms. Therefore, drugs targeting the NO pathway carry an inherent risk of adversely affecting cardiovascular health.

Importantly, in recent years, the focus in drug development has shifted toward other mechanisms including glutamatergic modulators, such as NMDA receptor inhibitors (e.g., ketamine) and AMPA receptor potentiators. The glutamatergic synapse and mechanisms revolving around NMDA and AMPA receptors are by themselves important mediators of mental disorder pathology. Notably, NO signaling functions as a downstream mediator of synaptic plasticity and has also been suggested to be involved in effects of some of the glutamatergic modulators under clinical investigation (9).

Another mechanism by which NO may contribute to mental disorder pathology is via neuroimmune pathways. Inflammation is a critical mechanism contributing to pathology across many different mental conditions including schizophrenia, depression, and bipolar disorder (10). NO synthesis can be triggered by inflammation-induced activation of iNOS providing another pathway by which NO may interfere with mental disorders, possibly also through DDAH/ADMA regulation (4).

The hype around NO in mental disorder pathology that started in the late 1990s and carried into the 2010s has somewhat cooled after early promising preclinical evidence for the treatment potential of NO modulators did not translate into meaningful clinical outcomes. However, NO is far from a lost cause, and we are now seeing the final phase of the hype cycle commonly referred to as the plateau of productivity. There is still a steady output of studies revolving around NO function in psychiatry, and we are getting a clearer picture by the day of the many different mechanisms contributing to this molecule’s function (see Figure 1). The mechanisms exerted by DDAH (4) are one such example, adding another piece to the puzzle of NO-related changes in mental illness. NO seems to interact with many mechanisms and pathways linked to mental disorder pathology and treatment. Therefore, NO could be considered a secret agent of pathology that affects or mediates mechanisms that are common across different disorders, both psychiatric and somatic. Whether this will eventually lead to the successful implementation of NO-based therapies remains to be seen. However, the advancements in mechanistic insight such as those presented in the review by Vareltzoglou *et al.* (4) further disentangle the complexity of mental disorder pathology and may enable more refined approaches to diagnosis and treatment.

**Figure 1:**
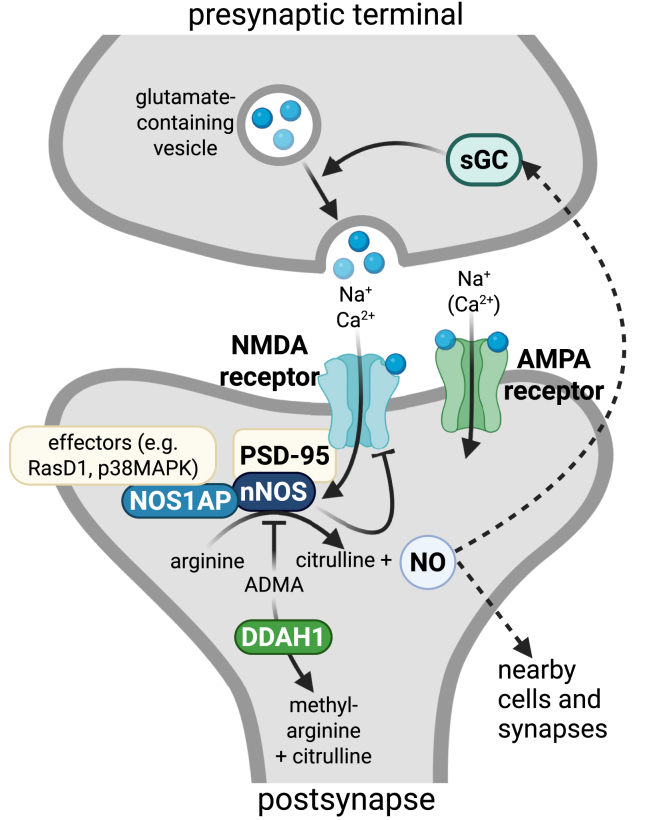
Schematic representation of nitric oxide (NO) pathways at the glutamatergic synapse. Dysbalanced homeostasis may lead to mental disorder pathology. (Figure created in BioRender) ADMA, asymmetric dimethylarginine; DDAH1, dimethylarginine dimethylaminohydrolase 1; nNOS, neuronal nitric oxide synthase; PSD, postsynaptic density; sGC, soluble guanylate cyclase.

## References

[1] Culotta E., Koshland D.E. (1992). NO news is good news. Science.

[2] Andrabi S.M., Sharma N.S., Karan A., Shahriar S.M.S., Cordon B., Ma B., Xie J. (2023). Nitric oxide: Physiological functions, delivery, and biomedical applications. Adv Sci (Weinh).

[3] Freudenberg F., Alttoa A., Reif A. (2015). Neuronal nitric oxide synthase (NOS1) and its adaptor, NOS1AP, as a genetic risk factors for psychiatric disorders. Genes Brain Behav.

[4] Vareltzoglou M.R., Rodionov R.N., Vernon A.C., Bernhardt N. (2025). The emerging role of the DDAH proteins in psychiatric disorders. Biol Psychiatry Glob Open Sci.

[5] Nair P.C., Mangoni A.A., Rodionov R.N. (2024). Redefining the biological and pathophysiological role of dimethylarginine dimethylaminohydrolase 2. Trends Mol Med.

[6] Carlström M., Weitzberg E., Lundberg J.O. (2024). Nitric oxide signaling and regulation in the cardiovascular system: Recent advances. Pharmacol Rev.

[7] Alda M. (2019). Methylene blue in the treatment of neuropsychiatric disorders. CNS Drugs.

[8] Zoupa E., Pitsikas N. (2021). The nitric oxide (NO) donor sodium nitroprusside (SNP) and its potential for the schizophrenia therapy: Lights and shadows. Molecules.

[9] Freudenberg F., Reif-Leonhard C., Dawson G.R., McKernan R.M., Reif A. (2025). All roads lead to glutamate: NMDA and AMPA receptors as targets for rapid-acting antidepressants. Pharmacol Res.

[10] Bauer M.E., Teixeira A.L. (2019). Inflammation in psychiatric disorders: What comes first?. Ann N Y Acad Sci.

